# Experimental Investigation of Cumulative Damage and Self-Healing Properties of Smart Cementitious Composite under Continuous Compression Load

**DOI:** 10.3390/ma16186090

**Published:** 2023-09-06

**Authors:** Weihong Chen, Chunhui Han, Yi Liu, Kai Feng, Shusen Zhuang

**Affiliations:** 1 College of Civil Engineering, Fuzhou University, Fuzhou 350116, China; chenwh@fzu.edu.cn (W.C.); liuyi610@126.com (Y.L.);; 2 School of Advanced Manufacturing, Fuzhou University, Jinjiang 362251, China

**Keywords:** sustained compressive loading, water absorption, loading level, self-repairing, engineered cementitious composites

## Abstract

This study investigated the effect of sustained loading on the cumulative damage of a newly developed smart cement-based self-healing composite material (SMA-ECC). SMA-ECC is composed of engineered cementitious composite (ECC) and shape memory alloy (SMA) fibers. A uniaxial compressive test with five predefined loading levels (0%, 30%, 40%, 50%, and 60% of compressive strength) was conducted on SMA-ECC hollow-cylindrical specimens and ECC control hollow-cylindrical specimens. The cumulative damage was mainly determined by changes in the total water absorption of different groups of specimens during three different periods (not loaded, at a predefined loading level, and after unloading). A normalized water content index was proposed to couple the effects of self-healing, sustained loading, and cumulative damage. The test results indicate that the cumulative water absorption of SMA-ECC was 35% lower than that of ECC, which may indicate less irreparable damage. In addition, the self-healing ability of SMA-ECC specimens under different compression load levels was evaluated through normalized water content analysis. SMA-ECC exhibited a 100% repair rate at load levels of 30% and 40%. At a higher load level of 60%, the repair rate of SMA-ECC was 76%. These results collectively emphasize the significant impermeability and self-healing performance of SMA-ECC after unloading.

## 1. Introduction

Throughout the lifespan of reinforced concrete structures, external factors such as the impact of ship hulls, waves, and intense sunlight can lead to damage [1,2,3]. These defects provide easy access for seawater to enter the inner part of the reinforced concrete structure [4,5,6]; thus, corrosion of the rebar starts once the chloride content reaches its upper limit, and after that, corrosion products pile up and harm the interface between rebar and concrete cover, which causes irreversible damage [7,8,9,10,11]. This sequence of events significantly impacts the overall durability of reinforced concrete structures. To obtain better durability of the reinforced concrete structures in marine environments, it is worth investigating ways to reduce its cumulative damage.

Our simulated experimental setup was mainly based on the marine environment; thus, cumulative damage was evaluated through a water absorption index based on related research [12,13,14]. The water absorption was determined by capillary absorption, since the damaged reinforced concrete structures in service were mainly under partially saturated environments, especially wet–dry areas like splash zones and tidal zones [15,16,17]. Current research has mainly focused on the quantification of capillary absorption; the effect of loading is not considered. However, there is evidence that loading has a noticeable influence on capillary absorption [18,19,20], especially when the loading level is 70~90% of the ultimate compressive strength. Additionally, existing theoretical prediction models of capillary absorption have merely considered a single factor into account, even though the practical engineering environment is affected by mechanical and environmental factors.

As aforementioned, loading has a strong effect on capillary water absorption. Researchers have investigated the effect of loading by creating prefabricated cracks on test specimens and then measuring the water absorption content. It is reported that there is a negative impact of increasing crack width in terms of capillary water absorption [21,22,23]; wider cracks result in a higher water absorption content. To simulate the different damage conditions, researchers have applied changing loading levels to the water absorption specimens. It is proven that a certain relation exists between the loading levels (i.e., damage condition) and water absorption content, and reducing this damage would bring the benefit of reducing capillary water absorption. Concerning reducing damage, the concept of self-healing [24,25,26] has been proposed and developed for a decade, including in the authors’ previous research [27,28], which proposed a new smart cement-based material (SMA-ECC). The smart cementitious composite SMA-ECC is composed of engineered cementitious composite (ECC) and shape memory alloy (SMA) fibers. SMA fibers can recover plastic deformation, effectively mending millimeter-scale cracks in concrete [29]. Engineered cementitious composite (ECC), as a novel type of fiber-reinforced cementitious composite, exhibits noteworthy attributes involving generating multiple micro-cracks, while maintaining controlled crack widths [30], effectively addressing the limitation of SMA fibers in repairing only small-scale cracks. This new type of smart cement-based material is capable of closing cracks of less than 150 μm in size, and cracks of less than 50 μm can be fully closed. According to the research, beyond its addition for crack closure, the internal damage of the SMA-ECC also exhibited noticeable recovery from internal damage. Thus, utilizing the SMA-ECC in marine environments could be a promising solution for improving the corrosion resistance of cement-based structures.

In this regard, our research experimentally studied the capillary water absorption behaviors of SMA-ECC and ECC under sustained compressive loadings through an improved water absorption test. The effect of predefined compressive loading levels (*λ_c_* = 0, 30%, 40%, 50%, and 60%) on water absorption was investigated through capillary water absorption tests. Furthermore, to investigate the influence of load-induced damage on the behavior of water absorption, water absorption tests were conducted at three different stages (not loaded, at a predefined loading level, and after unloading), and the cumulative water content and water absorption capacity were measured. The damage-repairing ability was also evaluated in this paper through the change of water absorption of intact and cracked SMA-ECC specimens.

## 2. Experimental Program

### 2.1. Materials, Mix Proportions, and Mechanical Properties

The mix proportions of SMA-ECC and ECC are listed in Table 1. The raw materials used in the SMA-ECC mixture were grade 42.5 ordinary Portland cement, I-grade fly ash with a mesh of 5000, S95 slag power with a mesh of 1000, silica sand, water, polyvinyl alcohol (PVA), shape memory alloy (SMA) fibers, and a high-range water-reducing admixture (HRWRA). The particle size of silica sand ranged from 100 mesh to 325 mesh (i.e., 150~45 µm). The geometrical and mechanical properties of PVA and SMA fibers are listed in Table 2. In addition, a heat treatment process was used to improve the super-elastic property of SMA, as described previously by the authors [28].

The mechanical properties of SMA-ECC and ECC at 28 days are given in Table 3. The compressive strength and elastic modulus were determined from axial compressive tests on 100 mm × 100 mm × 100 mm cubes and 100 mm × 100 mm × 300 mm prisms, respectively, according to the Chinese standard of GB/T 50081-2019 [31]. The flexural strengths were acquired with four-point bending tests on a prismatic specimen of 100 mm × 100 mm × 400 mm. Each group included three specimens.

### 2.2. Specimen Design and Preparation

**Figure 1:**
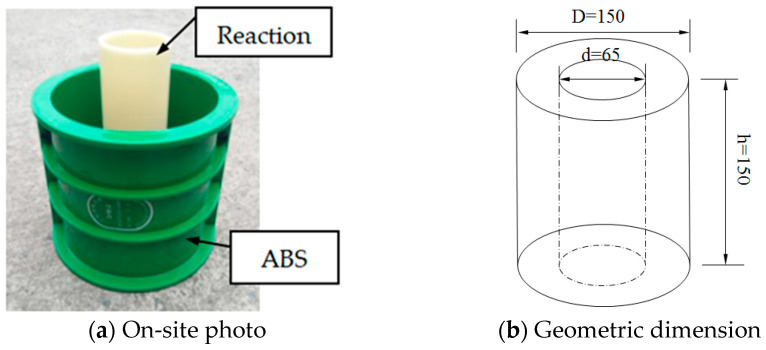
Design and size of the hollow-cylindrical specimen.

A total of 30 hollow-cylindrical specimens were prepared and divided into two categories: half SMA-ECC and the other half ECC. ECC specimens served as control specimens for the SMA-ECC ones. Figure 1a,b shows the design of the hollow-cylindrical specimen, which aimed to achieve synchronous coupling of uniaxial compression and water absorption. Both surfaces of the specimens were smoothed for better contact between the specimens and the loading setup. All specimens were immersed in lime-saturated water for 60 days to ensure complete hydration of the cement matrix. Prior to the capillary water absorption test, the water-saturated specimens were dried in a thermostat. Two annular rubber pads with a thickness of 10 mm were fixed on the ends of the specimen using glass glue to prevent water leakage during the test.

**Figure 2:**
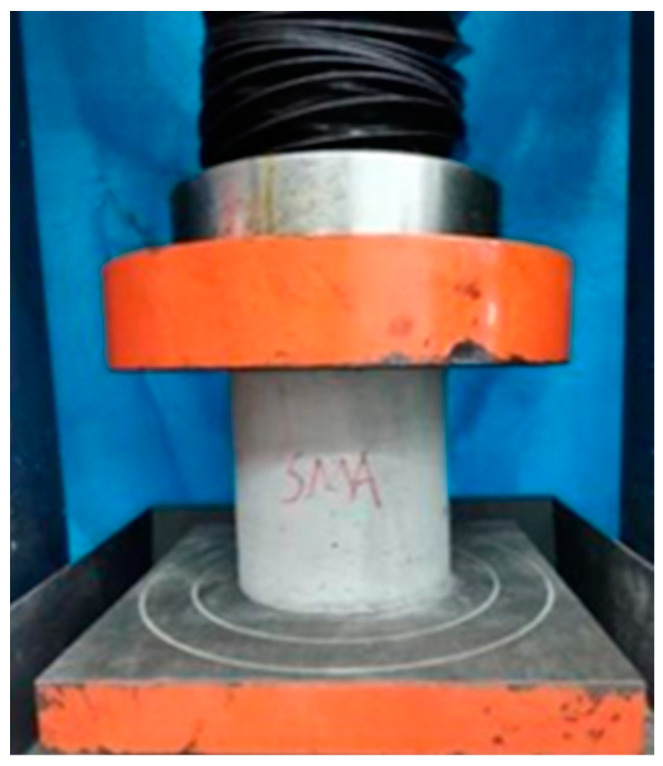
Compression test of hollow cylinder.

Compression tests were conducted on the hollow-cylindrical specimens, as illustrated in Figure 2. Three specimens were tested for each group, and the average value was considered as the final result for the ultimate compressive strength. The results showed that the ultimate compressive strength of SMA-ECC and ECC were measured at 50.3 MPa and 53.3 MPa, respectively. Each category of specimens consisted of five groups, with each group containing three identical specimens. The first group of specimens in each category was used to measure water absorption with an intact matrix. These specimens were then loaded monotonically until failure to determine the ultimate compressive strength. The specimens of the rest groups were loaded to 30%, 40%, 50%, and 60% of *f_c_*, where *f_c_* is the ultimate compressive strength of the intact hollow-cylindrical specimens. Details of the loading specimens are presented in Table 4.

### 2.3. Test Setup for Coupled Sustained Loading and Water Absorption

The traditional gravimetric water absorption measurement method proposed in ASTM C1585 [32] has inherent issues as it requires moving the test specimens out of the water several times to measure weight increments. Therefore, a specially designed test setup was adopted in this study to achieve the synchronous coupling of sustained loading and water absorption.

**Figure 3:**
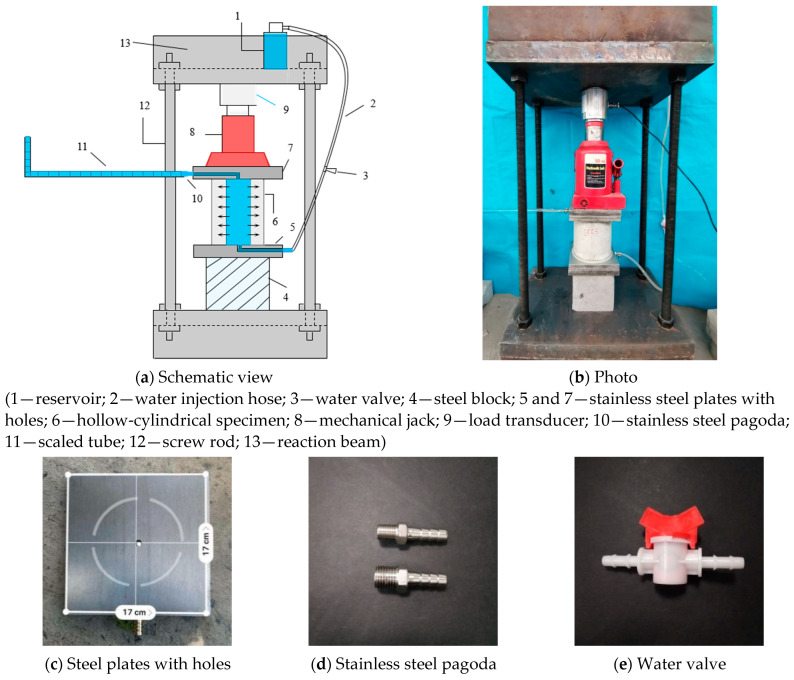
Test setup and equipment of capillary water absorption under sustained compressive loading.

The novel device consists of five parts: a water supply unit, horizontal observation unit, water absorption unit, loading measurement unit, and reaction force unit. The water supply unit includes a reservoir, water valve, and water injection hose. A horizontal tube with a scaled ruler serves as the observation unit to measure the cumulative water content absorbed by the specimen. Two stainless steel plates with holes are placed on the top and bottom of the specimen to connect the scaled tube and water injection hose, which have inner diameters of 6 mm each. The design of this test setup is shown in Figure 3.

The water content loss due to specimen absorption is negligible since water-filling time was very short (less than 25 s). Thus, it is assumed that the cumulative water content is measured immediately after the hollow-cylindrical specimen’s cavity is completely filled.

### 2.4. Loading and Testing Procedures

Four different predefined loading levels were set in this experiment, i.e., 30%, 40%, 50%, and 60% of the compressive strength of the materials. Three hollow-cylindrical specimens were employed at each loading level to study capillary water absorption across three stages (before loading, at a predefined loading level, and after unloading). The specimens before loading were treated as the control group compared to the specimens under sustained loading.

The predefined loading level (*λ_c_*) is defined by Equation (1).

(1) $$\lambda_{c}=\frac{\sigma_{c}}{f_{c}}\times 100\%$$

where *σ_c_* is the applied stress(MPa), *f_c_* is the ultimate compressive strength of the SE-1 and E-1 specimens (MPa). The details of the loading levels are presented in Table 4.

**Figure 4:**
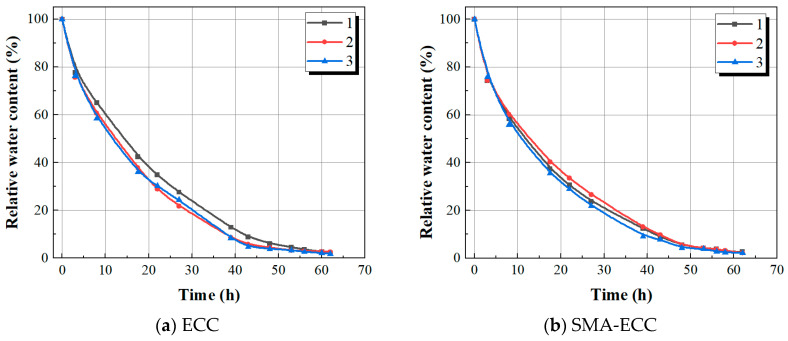
Isothermal drying curve of hollow-cylindrical specimens.

Prior to the water absorption test, all specimens were dried in a thermostat at a consistent temperature of 80 °C until a steady mass was achieved. The relative water content *θ* of hollow-cylindrical specimens can be determined by Equation (2).

(2) $$\theta =\frac{m_{i}-m_{d}}{m_{s}-m_{d}}\times 100\%$$

where *m_i_* is the actual mass of the specimen at a certain time (g); *m_d_* is the mass of the specimen in full dryness; and *m_s_* is the mass of the specimen in water saturation. Figure 4 shows the variation of relative water content in specimens. It can be seen that the mass of specimens decreased to a constant value at a drying time of about 60 h. The dried specimens were stored in a desiccator at a room temperature of 25 °C until the test began.

The specimen was gradually loaded to the predefined loading level and maintained under that load. Then, the hollow space of the specimens was filled with water obtained from an outer water tank. After overflowing through the scaled tube, the water valve was closed and the initial position of water in the horizontal scaled tube was immediately recorded. The change in the position of water in the scaled tube was directly recorded at various time intervals (5, 10, 20, 30, 60, 120, and 180 min) during the 9 h testing period, which determined the cumulative water content absorbed by the specimen.

Additionally, to investigate the effect of imposed load damage on the capillary water absorption of SMA-ECC, two-stage water absorption tests (i.e., without loading and after unloading) were conducted on the undamaged and pre-damage state of the SMA-ECC, respectively, following the aforementioned testing procedures.

### 2.5. Estimation of Cumulative Water Absorption

For a porous cement-based material, the cumulative content of water absorption per unit cross-section caused by capillary action can be determined by Equation (3) [32]:

(3) $$i=\frac{\Delta V}{A_{c}}=\frac{\Delta_{m}}{\rho_{w}A_{c}}=S\sqrt{t}+b$$

where *i* is the cumulative content of absorbed water per unit area (mm); Δ*V* is the volume of absorbed water (mm^3^); *A_c_* is the cross-sectional area of water absorption in the specimen (mm^2^); Δ*_m_* is the mass of absorbed water (g); *ρ_w_* is the density of water (g/mm^3^); *t* is the water absorption time (minutes); *S* is defined as the sorptivity, describing the rate of water absorption in porous cement-based materials; and *b* is a correction factor for considering the rapid water absorption when the specimen is initially in contact with water.

**Figure 5:**
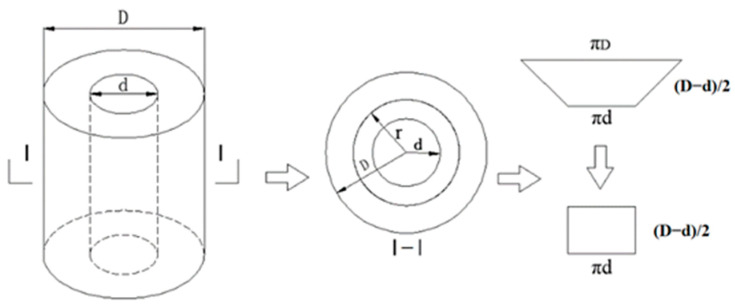
Schematic view of simplified absorption area.

The hollow-cylindrical specimens experience dynamic changes in the front area of water ingress as water is transported radially. To facilitate the application of Equation (3), simplification of the varying absorption area of the hollow-cylindrical specimen can be achieved [33], as shown in Figure 5. Assuming that the total porosity of the specimen is filled with water up to the saturated water level, the mass of absorbed water can be written as follows:

(4) $$\Delta m=pV_{c}\rho_{w}=p\pi \left(r^{2}-d^{2}/4\right)h\rho_{w}$$

(5) $$r=\sqrt{\frac{1}{4}d^{2}+\frac{d_{0}^{2}\cdot \Delta l}{4\rho_{w}\cdot p\cdot h}}$$

(6) $$p=p_{0}{\left[\frac{E_{b}\left(1-p_{0}\right)+2\left(1+\nu_{b}\right)\left(1-\nu_{b}\right)q_{b}}{E_{b}\left(1-p_{0}\right)+\left(1+\nu_{b}^{2}\right)\left(1-2\nu_{b}+p_{0}\right)q_{b}}\right]}^{2}$$

where *p* is the effective porosity of the specimen subjected to loading; *r* is the average radius of water penetration depth at a given time Δ*t* (mm); *d* is the inner diameter of the hollow-cylindrical specimen (mm); *d*_0_ is the inner diameter of the scaled tube (mm); *h* is the height of the specimen (mm); Δ*l* is the reduced water length in the scaled tube at a given time Δ*t* (mm); *p*_0_ is the initial porosity of the specimen without loading; *E_b_* and *ν_b_* are Young’s modulus and Poisson’s ratio of the porous materials, respectively; and *q_b_* is the external equivalent stress (i.e., the compressive stress on the specimen in this study). Based on the above assumption, the correction factor *β* considering the deviation caused by the simplified absorption area can be defined as follows:

(7) $$\beta =\frac{\Delta m}{\Delta m_{s}}=\frac{r+0.5d}{d}$$

In summary, the cumulative content of water absorption *i* can be finally calculated as

(8) $$i=\frac{\Delta m_{s}}{\rho_{w}\cdot \pi d\cdot h}=\frac{\Delta m}{\beta \cdot \rho_{w}\cdot \pi d\cdot h}=\frac{d_{0}^{2}\cdot \Delta l}{4\beta \cdot d\cdot h}$$

To minimize the effect of uncertain factors, such as unmeasured initial water content absorbed during the injection process and variations in pore structures among specimens, the averaged values of three specimens for each target loading level were used as the test results.

## 3. Results and Discussion

### 3.1. Cumulative Water Content under Sustained Compressive Loading

**Figure 6:**
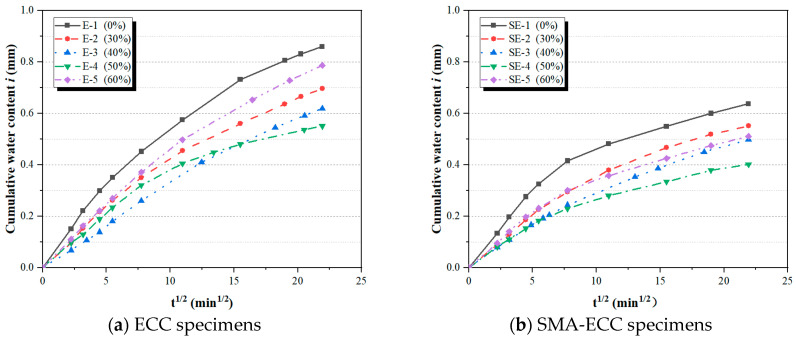
Cumulative water content of all specimens under target compressive loads.

Figure 6 displays the typical results of cumulative water content *i* of all specimens subjected to predefined loading levels (i.e., 0, 0.3, 0.4, 0.5, and 0.6 *f_c_*). It can be found that compressive loading has an apparent impact on capillary water absorption. With an increase in the loading level, the cumulative water content initially decreases and then increases beyond the critical loading level at a given absorption time. It is reported that this critical loading level is often associated with the effect of micro-cracks induced by loads on the permeability of cement-based materials [19]. In general, the crack propagation process of concrete subjected to sustained compressive loads contains three stages, the deformation of pore structure, the development of original micro-cracks, and the effect of micro-cracks bridging [29]. Compressive loads below the critical loading level have a ‘compacting effect’ on concrete, resulting in the partial closure of capillary pores and original micro-cracks, which further hinder the process of water transportation. As compressive loads exceed the critical loading level, the ‘micro-cracking effect’ plays a dominant role in facilitating the propagation of original micro-cracks and the development of new micro-cracks, inversely accelerating the rate of water transportation. Loo [34] reported that the micro-cracks effect, which determined the rate of water penetration into concrete, usually occurred at loading levels between 15% and 45%.

From Figure 6, it can be observed that both SMA-ECC and ECC specimens exhibit a ‘compaction effect’. As discussed previously, the compacting effect decelerates the water transportation process, while the cracking effect enhances it. Therefore, when the deterioration of the ‘cracking effect’ cannot be balanced by the enhancement of the ‘compacting effect’ on the impermeability of cement-based materials, the cumulative water content in the *i-t*^1/2^ curve will change from decreasing to increasing. The changing behavior of water absorption under compressive loading is similar to the behavior of chloride-invading concrete studied by Wang et al. [35]. In the loading range from 0% to 50% *f_c_*, the cumulative water content of both SMA-ECC and ECC specimens gradually decreases with an increase in the loading level. When the compressive stress reached a critical loading level (i.e., 0.5 *f_c_*), the cumulative water content of SMA-ECC and ECC specimens exhibited an increasing trend as the loading level further increased. However, despite having similar critical stress levels, SMA-ECC consistently demonstrates lower cumulative water absorption compared to ECC across the five stress levels. This indicates that SMA-ECC materials exhibit an excellent ability to maintain good impermeability at higher compressive loading levels.

**Figure 7:**
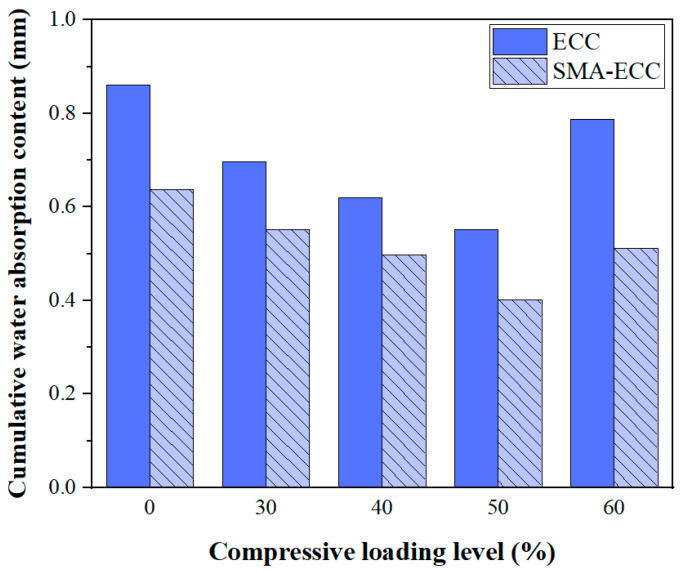
Cumulative water absorption content of specimens at various loading levels.

**Figure 8:**
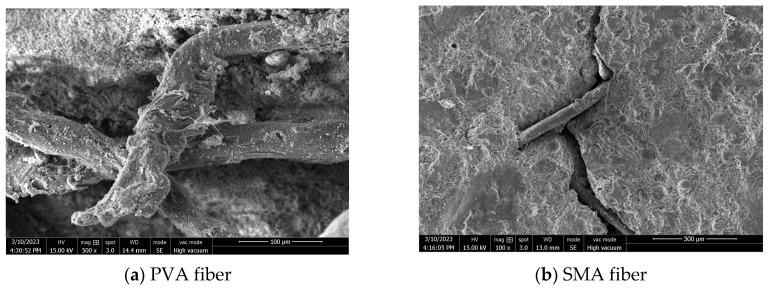
SEM on PVA and SMA fibers of SE-1.

The cumulative water absorption content of ECC and SMA-ECC hollow-cylindrical specimens within a given water absorption time of 520 min is shown in Figure 7. It is evident that ECC specimens exhibit significantly higher water absorption compared to SMA-ECC specimens across all loading levels of 0~60%. Notably, at a load level of 60%, the cumulative water absorption of SMA-ECC is 35% lower than that of ECC. The water absorption behavior is closely associated with the composition, pore structure, and porosity of cement-based materials. However, as indicated by Table 2, there is little difference in the initial porosity between ECC and SMA-ECC. This suggests that the presence of SMA fibers contributes to a supplementary closure of existing cracks. It is noteworthy that ECC specimens exclusively incorporate PVA fibers, characterized by their relatively diminutive dimensions and limited length, rendering them susceptible to extraction from cracks. Compared with ECC, SMA-ECC has two types of fibers and lower initial porosity. Moreover, PVA and SMA fibers have a greater ‘bridging effect’ on cracks, inhibiting their development and propagation. This is demonstrated in the scanning electron microscopy (SEM) image of Figure 8, where bridging of the SE-1 with SMA fibers after the water absorption test was observed.

### 3.2. Sorptivity under Sustained Compressive Loading

Sorptivity, a vital parameter for assessing the fluid transportation capability of unsaturated porous cement-based materials through capillary forces, is well recognized [36]. The cumulative water content (*i-t*^1/2^) curve features two linear segments corresponding to the initial and secondary sorption stages. These stages can be attributed to distinct water transportation mechanisms: the initial stage is governed by the swift saturation of capillary pores, while the secondary stage arises from the gradual filling of air voids or micro-cracks [37]. Notably, the *i-t*^1/2^ curves (Figure 6) distinctly demonstrate a pivotal turning point approximately 120 min into the process. According to Equation (1) and ASTM C1585 [32], the initial sorptivity *S*_1_ and secondary sorptivity *S*_2_ are defined as the first and second slopes of the two linear portions in this curve, respectively. The initial and secondary sorptivity for all specimens at various compressive loading levels are listed in Table 4.

**Figure 9:**
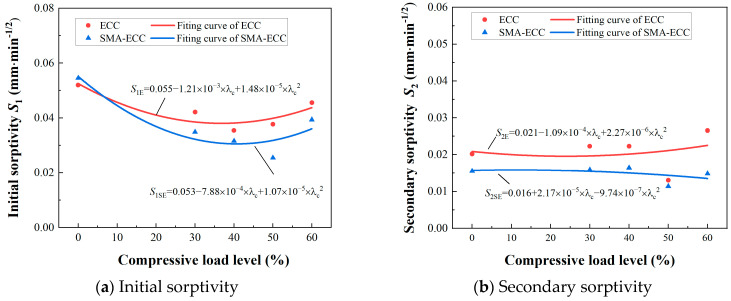
The sorptivity vs. compressive loading levels.

Figure 9 depicts the correlation between compressive loading levels and sorptivity for both concrete and SMA-ECC specimens. As evident from the graph, the initial water absorption rate surpasses that of the secondary stage, attributed to the swifter absorption through capillary pores compared to air voids in the materials. Additionally, as compressive stress levels increase, the initial sorptivity *S*_1_ diminishes until reaching a critical point, followed by a slight increase. Conversely, the secondary sorptivity *S*_2_ of SMA-ECC specimens remains relatively stable. Throughout continuous compressive loading, the relationship between initial and secondary sorptivity and loading levels displays nonlinearity. Bao and Wang [29] indicated that fitting test data with Equation (9) can yield an approximate relationship between sorptivity and loading level for concrete.

(9) $$S=S_{0}+\alpha \lambda_{c}+\beta \lambda_{c}^{2}$$

where *α* and *β* are the experimental fitting parameters, and *S*_0_ is the sorptivity of the hollow-cylindrical specimens without loading for ECC and SMA-ECC.

It can be observed that at the loading levels, the sorptivity of SMA-ECC specimens is significantly lower than that of ECC specimens. For example, the initial sorptivity of Specimen SE-5 is about 13.8% lower than that of ECC at 60% loading level. This phenomenon can be attributed to the superior crack self-control ability of SMA-ECC materials, which maintain a maximum crack width of below 100 µm under loading [28]. This characteristic facilitates the more efficient filling of fine cracks with particles generated by subsequent hydration reactions.

The average value of *S*_1_ and *S*_2_ is considered as the average sorptivity. In order to highlight the superior impermeability of SMA-ECC compared to ECC, the average sorptivity of ECC is considered as the relative condition, and the relative average sorptivity *η* is defined. The calculation equation is as follows:

(10) $$\eta =\frac{S_{SE}}{S_{E}}\times 100\%$$

where, *S_SE_* represents the average sorptivity of SMA-ECC (mm∙mm^−1/2^), and *S_E_* represents the average sorptivity of ECC (mm∙mm^−1/2^).

**Figure 10:**
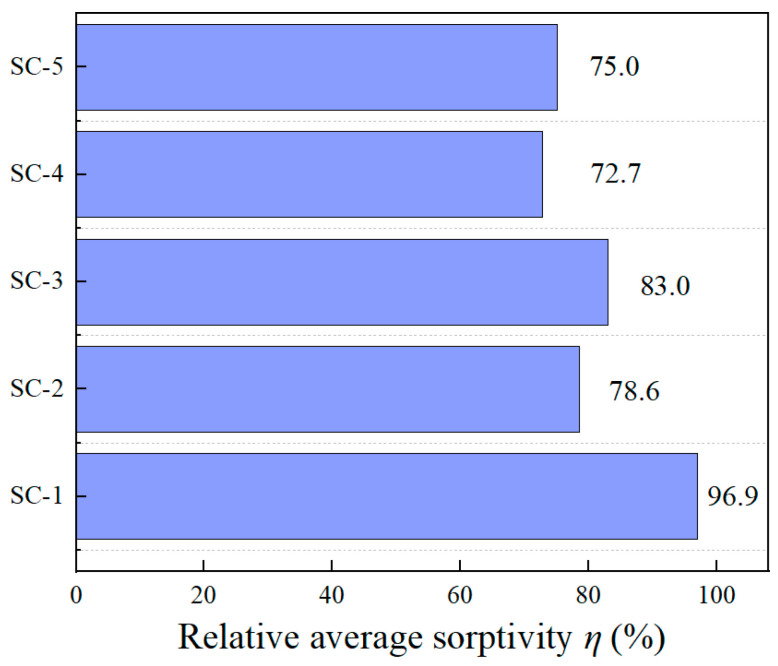
Relative sorptivity at various compressive loading levels.

The comparative analysis of the relative average sorptivity of SMA-ECC is illustrated in Figure 10. Both SMA-ECC and ECC exhibit similar water absorption rate responses to varying stress levels. Specifically, as the stress levels increase from 0 to 30%, from 30% to 50%, and further from 50% to 60%, the relative average absorption rates follow a pattern of initial increase, subsequent decrease, and then another increase. In their undamaged states, SMA-ECC and ECC show similar average sorptivity. However, under damage conditions, the relative average sorptivity of SMA-ECC consistently remains lower than their undamaged counterparts. It is noteworthy that the trend of SMA-ECC’s sorptivity consistently remains lower than that of ECC. This can be attributed to the incorporation of SMA fibers, which potentially mitigate the early-stage shrinkage of the cementitious matrix, thereby reducing the formation of internal shrinkage cracks and pore networks. Consequently, this mechanism constrains the internal moisture transfer within the material.

### 3.3. Cumulative Water Absorption before and after Damage

**Figure 11:**
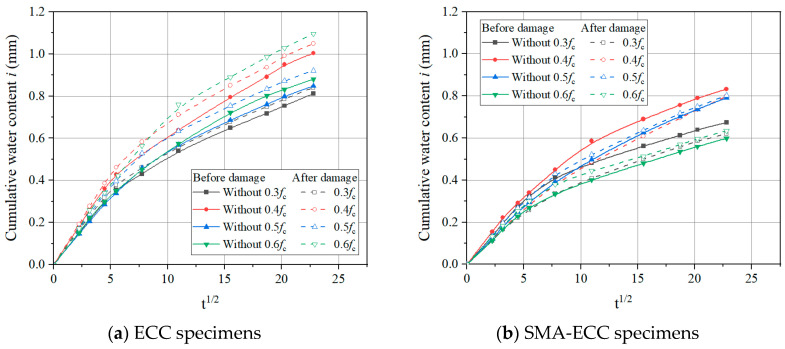
Cumulative water content before and after damage at target loads.

This section aims to investigate the effect of damage induced by sustained compressive loads on the capillary water absorption behavior of ECC and SMA-ECC specimens. Typical results of cumulative water absorption for ECC and SMA-ECC hollow-cylindrical specimens before and after damage induced by various target loads (0.3, 0.4, 0.5, and 0.6 *f_c_*) are shown in Figure 11, in which the state of before and after damage corresponds to two loading patterns (without loading and unloading after loading to target loading level). From Figure 11, within a certain water absorption test period, the cumulative water content of ECC specimens with imposed loading damage has an apparent increase compared to that of the same batch of specimens without damage, especially at a high loading level of 0.5 *f_c_*. And the cumulative water content of damaged specimens significantly increases with an increase in compressive loading level. This can be attributed to the proliferation and development of micro-cracks with increasing applied loading, which provides easy access for water transportation in the cement matrix. Across the predetermined compressive loading range of 30% to 50%, the cumulative water content of compromised SMA-ECC specimens is inferior to that of intact SMA-ECC. This outcome can be attributed to the activation of the ‘super-elastic effect’ inherent in SMA fibers, leading to the self-healing of cracks subsequent to unloading [37]. Specimen E-5 failed under a sustained loading of 0.6 *f_c_*, thus the water absorption properties at this loading level are not analyzed.

### 3.4. Self-Repairing Ability of SMA-ECC

The capillary water absorption test has been acknowledged as an effective method to assess the self-healing capacity of cement-based materials [30]. The self-repairing ability of SMA-ECC was evaluated by comparing the cumulative water content of ECC and SMA-ECC before and after damage induced by sustained compressive loading.

Based on the cumulative water content of intact specimens, the normalized water content *φ*(*t*) shown in Equation (11) is defined as the ratio of the cumulative water content of the specimens before and after suffering damage. The damage-repairing rate *R* of SMA-ECC is determined by Equation (12).

(11) $$\varphi (t)=\frac{i_{u}\left(t\right)}{i_{0}\left(t\right)}\times 100\%$$

(12) $$R=\frac{\varphi_{{}_{E}}-\varphi_{{}_{SE}}}{\varphi_{{}_{E}}-1.0}\times 100\%$$

where *i*_0_(*t*) and *i_u_*(*t*) are the cumulative water content before damage (i.e., not loaded) and after damage (i.e., after unloading), respectively; *φ_E_* and *φ_SE_* are the normalized water content of ECC and SMA-ECC at the end of water absorption test, respectively. If the value of *R* is greater than 1.0, it is considered as 100%.

**Figure 12:**
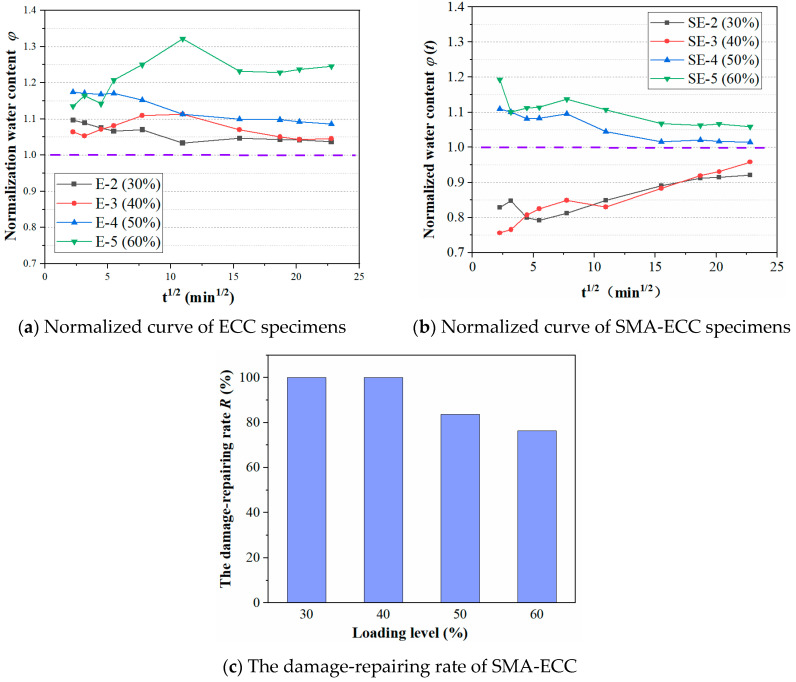
Self-repairing ability of ECC and SMA-ECC.

Figure 12a,b displays the normalized water content of ECC and SMA-ECC specimens at different compressive loading levels. The relative water absorption of ECC is greater than 1 in the range of 30~60% stress level, while the relative water absorption of SMA-ECC is less than 1 in the range of 30~40% stress level, and the relative water absorption of φ is about 1 in the range of 50~60% stress level. This indicates that after ECC is subjected to a certain load, part of the internal damage cannot self-heal, resulting in higher water absorption after unloading compared to the initial water absorption. SMA-ECC has excellent self-healing performance under flexural and tensile loads, and it can also reflect a certain self-healing ability under low loading levels, with better recovery of internal damage. Especially at 60% stress levels, the relative water absorption φ of SMA-ECC is much lower than that of ECC. This observation correlate well with the fracture self-healing properties discussed in Section 3.3.

Figure 12c displays the damage-repairing rate of SMA-ECC at target loading levels. The cumulative water content of the self-repaired specimens SE-2 and SE-3 with target loading of 0.3 *f_c_* and 0.4 *f_c_*, respectively, closely matches that of the intact specimens. The repairing rate of SMA-ECC reaches 100% at a loading level of 30% and 40%, and then decreases with an increase in loading levels. At a higher loading level of 60%, the repairing rate of SMA-ECC reaches only 76%. The results indicated that SMA-ECC has excellent self-repairing properties after unloading, which can be credited to the super-elasticity effect of the added SMA fibers. The stress-induced martensitic transformation mechanism provides a restoring force that partially self-closes micro-cracks when external loading is removed [27]. This self-closure process is influenced by the loading level, wherein the closure of cracks restricts the transport of external media such as water into the cement matrix, resulting in a decrease in water absorption content. As aforementioned, the optimal practical working loading level can be around 30% to 40% of the ultimate compressive strength of the specimens, with an upper limit of approximately 60% *f_c_*.

## 4. Conclusions

This paper presents an experimental study that examines the impact of sustained compressive loads and load-induced damage on the capillary water absorption of SMA-ECC, using an enhanced self-designed water absorption test setup. The evaluation of the self-repairing capability is performed by measuring the cumulative water content of SMA-ECC both with and without the influence of imposed load damage. The following conclusions are drawn based on the analysis of the experimental findings:

(1) With an increase in the studied compressive stress levels, the cumulative water content and initial sorptivity firstly decrease and then increase beyond the critical loading level at a given exposure time. The cumulative water absorption of SMA-ECC is consistently lower than that of ECC. In particular, at 60% stress levels, the cumulative water absorption of SMA-ECC is 35% lower than that of ECC.

(2) SMA-ECC exhibits better resistance behavior of water absorption under sustained compressive loading compared with ECC. The relative average sorptivity (ratio of ECC to SMA-ECC) was used to compare the impermeability of SMA-ECC and ECC. The relative average sorptivity can be maintained at about 75% under higher stresses such as 50% or 60%. This result can be attributed to the initiation of the inherent ‘super-elastic effect’ present in SMA fibers, which subsequently facilitates the self-repair of cracks upon unloading.

(3) In terms of the cumulative water content before and after damage, the normalized water content is proposed in this study. Based on the results of normalized water content, SMA-ECC exhibited an excellent damage-repairing ability below the loading level of 0.4 *f_c_*; however, the repairing rate is only 76% at a higher loading level of 0.6 *f_c_*.

These findings indicate that SMA-ECC has remarkable self-healing performance under damage. However, it is important to note that factors such as higher loading levels, duration of loading, and environmental conditions (including temperature and humidity) may also influence the water resistance properties of SMA-ECC. Moreover, the application of SMA-ECC in practical engineering components, such as marine structures like piles or bridge piers, merits investigation regarding its impact on structural mechanics and durability. Therefore, exploring the resistance to water penetration and self-healing capabilities of SMA-ECC in various environmental conditions, under different loading scenarios, and within actual structural elements presents a promising direction for further research.

## Figures and Tables

**Table 1:** Mix proportions of SMA-ECC and ECC (weight proportion).

	Cement	Fly Ash	Slag Powder	Silica Sand	Water	HRWRA	PVA (%V*_f_*)	SMA (%V*_f_*)
SMA-ECC	0.3	0.55	0.15	0.4	0.25	0.002	1.7%	0.7%
ECC	0.3	0.55	0.15	0.4	0.25	0.002	1.7%	-

**Table 2:** Geometrical and mechanical properties of PVA and SMA fibers.

Type	Diameter (mm)	Length (mm)	Tensile Strength (MPa)	Elongation (%)	Young’s Modulus (GPa)	Density (kg/m^3^)
PVA	0.04	12	1560	6.5	42.8	1300
SMA	0.6	16	895	38.0	41.0	6450

**Table 3:** Mechanical properties of SMA-ECC and ECC.

Materials	Compressive Strength (MPa)	Flexural Strength (MPa)	Elastic Modulus (GPa)	Poisson’s Ratio
SMA-ECC	62.4	3.06	19.1	0.18
ECC	68.1	2.21	20.3	0.19

**Table 4:** Specimen details.

Material	Specimen	Loading Levels *λ_c_* (%)	Applied Stress *σ_c_* (MPa)	Effective Porosity *p* (%)
SMA-ECC	SE-1	0	0	5.520
	SE-2	30	15.1	5.509
	SE-3	40	20.1	5.505
	SE-4	50	25.2	5.501
	SE-5	60	30.2	5.498
ECC	E-1	0	0	5.640
	E-2	30	16.0	5.628
	E-3	40	21.3	5.625
	E-4	50	26.6	5.621
	E-5	60	31.9	5.617

## Data Availability

The data presented in this study are available on request from the corresponding author.
